# Multiparameter analysis of small non-flying mammals’ response to forest restoration post-bauxite mining in eastern Amazonia

**DOI:** 10.1371/journal.pone.0315904

**Published:** 2025-01-24

**Authors:** Halícia Celeste, Paula Cristina Rodrigues Almeida-Maués, Alexandra Maria Ramos Bezerra, Maria Aparecida Lopes, Marlúcia Bonifácio Martins, Ana Cristina Mendes-Oliveira

**Affiliations:** 1 Laboratory of Ecology and Zoology of Vertebrates, Institute of Biological Science, Federal University of Pará, Belém, Pará, Brazil; 2 Museu Paraense Emílio Goeldi, Belém, Pará, Brazil; 3 Faculdade Estácio de Castanhal, Castanhal, Pará, Brazil; 4 Unama Parque Shopping, Belém, Pará, Brazil; Saint Xavier’s College, INDIA

## Abstract

Bauxite mining has been caused severe changes in the natural ecosystems of the Amazon, but the restoration of these areas is mandatory by federal law in Brazil. The recolonization of fauna is crucial to establishing the ecological functions of recovering forests, and the small nonflying mammals can stand out in this process. Assessing taxonomic and functional diversity parameters, we demonstrated that in the early stages of forest recovery post-bauxite mining, between 6 and 11 years, it is possible to restore approximately 45% of the richness of small non-flying mammal species from the original habitats, that in this case were altered Primary Forests. However, the species richness parameter alone does not reflect the recovery of taxonomic or functional diversity at this stage of forest succession. Although 34.8% of the species composition is shared between the Altered Primary Forest and Forest Areas in Restoration, the abundance distribution per species is less balanced in the latter habitat. The areas did not exhibit significant difference between the functional divergence and functional evenness of ecological functions performed by small nonflying mammals; however, they present differences in terms of the functional richness. We also observed that some functional traits of species, such as larger body mass, are more closely related to the structural characteristics of the Primary Forest, such as high basal area values, litter and percentage forest cover. In the forest recovery areas, we observed a predominance of terrestrial species and those with granivorous and insectivorous diets. Furthermore, our results highlight the importance of applying different taxonomic and functional diversity parameters to understand better the fauna recovery patterns in degraded areas undergoing restoration.

## Introduction

The Amazon’s contribution to the export of mineral resources in Brazil and worldwide is quite significant [1, 2]. Around thirteen regions of metallic deposits have strong mining potential in the Amazon, covering an area of approximately 1,110 km^2^ [3]. Currently, the gold extraction is responsible for 58% of this industry’s total in the Amazon region, followed by aluminium at 15%, tin at 13% and iron at 8% [3]. Bauxite, a mineral used to produce aluminium, is found mainly in plateau areas in the southeast region of the biome [4]. This mineral is concentrated in the soil surface layer, but its exploitation requires the complete removal of the forest cover and topsoil [1, 2]. To restore part of the soil microbiota and promote forest restoration, the sterile material resulting from mining has to be returned as a substrate for recomposing the exploited areas [3]. This material can be mixed with topsoil and receive chemical fertilization or be planted with native species [4]. However, it is unclear whether these processes are sufficient to allow the organisms to reestablish themselves during the recovery of this mined environment.

Restoration of the local fauna and flora should be part of ecological succession process in post-mining areas. In the initial stages of natural regeneration, the vegetation may have a predominance of pioneer species resistant to the sun that produce large amounts of seeds [5, 6]. In the tropics, between five and 20 years are considered the intermediate stages of succession [5], during which species composition may differ as a result of variable factors such as randomness [7], local environmental filters [8], limitation of dispersal [9, 10], and ecological interactions [11]. Subsequently, an increase in plant diversity and greater uniformity in litter distribution, may occur, generating microclimatic conditions more favorable to the colonization of a greater variety of soil invertebrates and small generalist vertebrate species [12–14]. These organisms in turn, will attract other vertebrate species along the course of succession, such as amphibians, reptiles, birds and other larger mammals [15]. In some induced cases of restoration of degraded areas, techniques are used to accelerate the forest recovery, including adding topsoil and sowing or transplanting seedlings (planting and nucleation). These techniques help overcome soil limitations for plant recruitment and survival, favouring dispersal and accelerating the succession process [20, 21]. In the case of the nucleation restoration process, the planting of woody plants functions as focal areas for the establishment of propagules, and can also be attractive to the fauna, such as birds and other taxa [22–25].The recolonization of terrestrial fauna in the intermediate stages helps define the course of restoring degraded areas [16]. It is important to emphasize that the success of this process does not depend only on the recolonization of plant species but also on a set of interspecific interactions involving fauna, including pollination, predation and seed dispersal, nutrient fixation and cycling [16–18]. In this context, the fauna recolonization allows positive feedback for the restoration of vegetation and vice versa.

However, the paths of fauna recolonization in degraded areas only sometimes follow a classic successional sequence [19, 20]. Restoration can often go through multiple states of balance, for example, in areas with numerous trajectories of degrading effects [21, 22]. Another option is the persistent non-equilibrium states, called stochastic, which assume the unpredictability and the role of external factors acting in the ecosystem [23, 24]. In this way, several factors can influence the trajectory of forest restoration, highlighting the type of disturbance, the intensity, and the duration of the disorder, in addition to the remaining biological legacy, making the structure of natural communities unpredictable in space and time [23, 24]. Another issue to be considered is that land-use changes normally lead to the loss of species and ecological traits that directly affect key ecological functions. Besides, changes in environmental conditions after disturbance can act as environmental filters, selecting a set of functionally similar species [25, 26]. Thus, studies that seek to understand the faunal recovery process in degraded areas must consider both the taxonomic and functional diversity of the fauna.

Delimiting trends in the functional diversity of a community in recovery allows for better adaptive management of restoration programs [48]. Increasing functional diversity to taxonomic diversity results in a more complete assessment of restoration progress [43, 44]. This approach investigates the structure of functional traits within a community and which traits are filtered or selected by the environment and their possible consequences on ecosystems [45–47].

Between the groups of fauna affected by land use changes, we highlighted the mammals, as they are more sensitive to environmental changes and responsible for performing critical ecological papers in the ecosystem, such as the top-down effect, that control the density of animal and plant populations [27, 28]. The absence of large-sized mammals, such as herbivores and big predators in an area, may cause trophic cascade effects [29], affecting plant recruitment and favoring small mammals’ outbreaks and medium-sized carnivore populations [30, 31]. In these cases, the ecological restoration act looks for the recovery or maintenance of threatened and native populations, as well as ecosystem structure and function [42].

Small non-flying mammals are among the mammal species with a high potential for environmental responses that can provide important information in tropical forest restoration processes, especially in the initial and intermediate stages. This group includes small rodents from Cricetidae and Echimyidae of the order Rodentia, and marsupials from the family Didelphidae, order Didelphimorphia [32]. These animals have a small body mass and great reproductive capacity, allowing faster responses to environmental changes. Furthermore, they play essential roles in ecosystems [33–35] controlling seed recruitment, allowing the entry of new species [36] and seed dispersal, especially in the stages of succession [37–39]. They are also crucial in the dynamics of soil transformation through their digging activities, whether to make burrows or nests, but also to bury seeds, and they act as essential predators of invertebrate populations and as dispersers of mycorrhizal spores [40, 41].

Vegetation structure significantly influences the occurrence, distribution, and habitat use of small, non-flying mammals [42]. Studies indicate that variables such as vertical structure of the vegetation, levels of canopy openness, and vegetation cover, and litter amount can act as determinants in structuring the assemblage of small mammals in tropical forests [43–45]. Vegetation structure influences the availability of food and shelter resources and can affect this fauna group’s taxonomic and functional diversity[43, 46, 47].

It is essential to highlight that species richness patterns tend to be more easily reestablished during the forest recovery process, considering that the small mammals can be one of the first groups of vertebrates to return to recovering areas, as many species are generalists and opportunists [48]. In contrast, species composition and abundance can change throughout the restoration process [49, 50]. Composition and abundance patterns, which define taxonomic diversity, can affect the functional diversity of areas under recovery [51–53]. A study in areas with different levels of forest restoration in Australia highlighted changes in the composition of the mammalian community, reflecting functional differences [54]. In the initial stages of restoration, invasive, herbivorous, terrestrial, generalist and open area species were observed, whereas in more advanced stages of restoration, endemic, folivorous, arboreal and fossorial species were established [54]. Another study analyzed the influence of the vegetation structure of restored areas on the taxonomic metrics of small mammals in the Dakota region of the USA [72], and the authors emphasize that the species composition in restored pastures, with low seedling diversity, differs from reference areas.

The combination of taxonomic and functional metrics in fauna studies is relatively recent, especially in the Amazon. Taxonomic groups such as birds, bats, small mammals, and lizards were targets of this multiple approach in the Amazon biome, using different anthropogenic approaches [73, 75]. In a global assessment, which does not include the Amazon fauna, with smaller mammal species, the authors observed that various functional groups present different recovery trajectories in post-mined recovery areas [76]. In this way, the complementary use of functional metrics in conjunction with taxonomic metrics can effectively verify the effects of anthropogenic disturbances and the effectiveness of restoring these environments after significant disturbances [35, 77]. In this research we investigated the taxonomic and functional response of the assemblage of small Amazonian mammals to the natural forest regeneration process after bauxite mining. We evaluated the response of the fauna of small non-flying mammals in areas of forest recovery in initial stages of ecological succession, compared to areas of primary forests in the eastern Amazon. We evaluate the effects of forest restoration, considering responses based on taxonomic diversity metrics, including richness, composition and abundance of small mammal species, and functional metrics. Furthermore, we verified the importance of the environmental variables that characterize the sampled habitats, in the differences in responses of the small mammal fauna, both with taxonomic and functional metrics. The general idea of the work is to show how a specific group of Amazonian vertebrate fauna, with a high potential for environmental response, is capable of presenting different results in the recovery process of post-bauxite-mined areas. Considering multiple diversity parameters, it is possible to choose small non-flying mammals as model organisms in forest recovery monitoring processes in areas at initial stages.

## Materials and methods

### Study area

**Fig 1:**
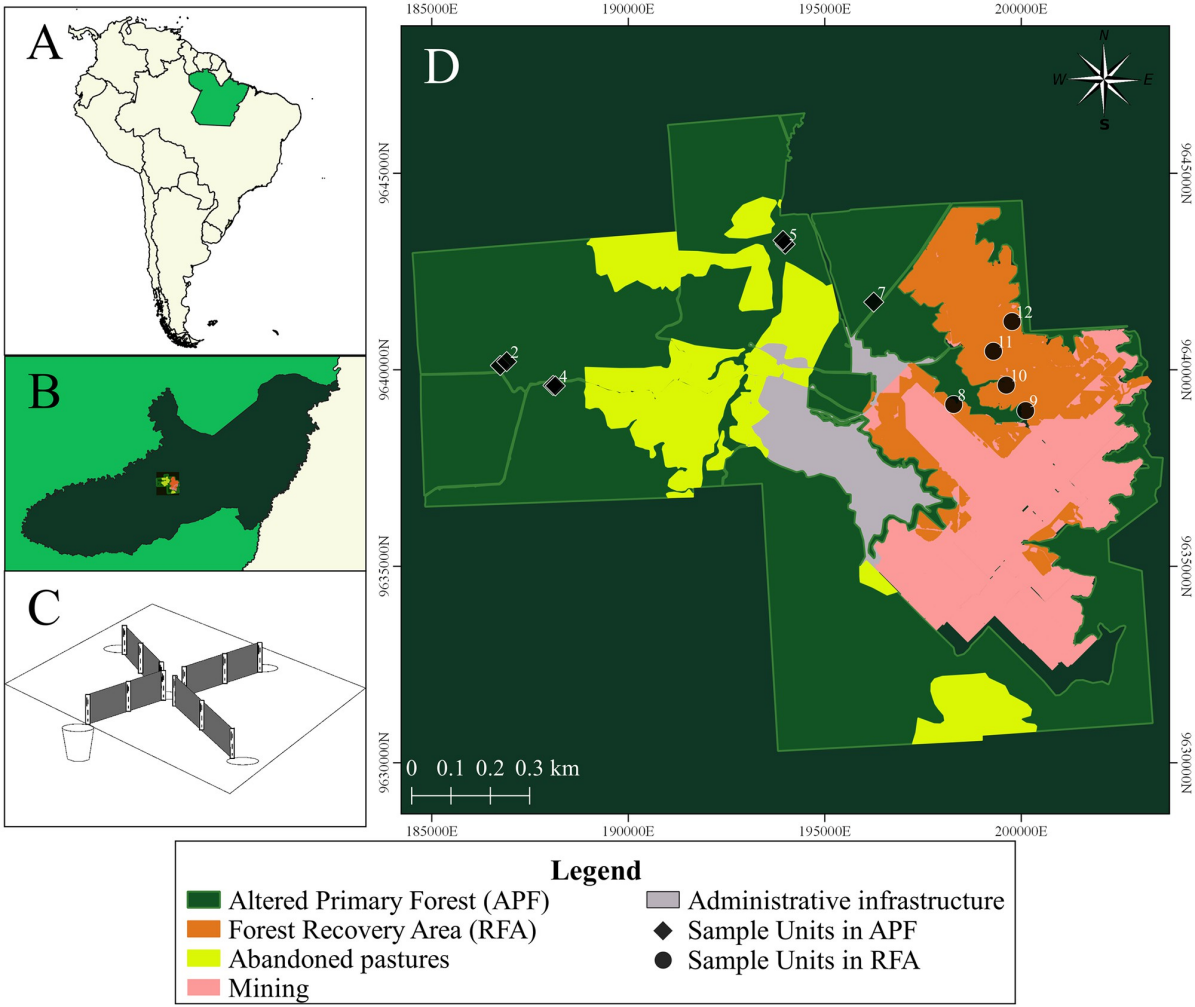
Study area map. Location of the study area highlighted in (A) South America in light gray, Brazil in yellow and the state of Pará in green; (B) municipality of Paragominas in dark green, highlighting the study area in the square in black; (C) Pitfall collection station model implemented at each sampling point; (D) Boundaries of the study area and the spatial distribution of sampling points in different habitats.

We conducted our study in the bauxite mine area of the Norsk Hydro Company, in the municipality of Paragominas, in the southeastern part of the State of Pará, in the Brazilian Amazon (Fig 1). The original predominant vegetation of this entire region was the Dense Ombrophylous Forest of Terra Firme [55]. However, the municipality of Paragominas has suffered an intense process of forest degradation and deforestation, especially since the 1970s [56]. Initially, both conventional and predatory logging impoverished the forests, and later agroindustry and livestock drastically reduced the forest areas in the region [57]. Paragominas lost about 28% of its native vegetation cover [58], 60% of the remaining forests in this region have already suffered some anthropogenic impact [59, 60], accumulating, between the years 2008 to 2022, 559 km^2^ of deforested area [61]. Thus, most of the forest remnants in the region are currently constituted by altered primary forests [62]. Specifically, the primary forests sampled in this study underwent selective logging in the 1980s and 1990s, resulting in some clearings with characteristics of secondary forests. However, despite signs of altered forest, their overall structure still retains the characteristics of conserved primary forest. In a floristic survey, Cerqueira et al. 2022 [63] recorded over 90 shrub species and 14 liana species in 9 one-hectare plots of forest in the same sampling areas as this study. Therefore, we refer to these areas as Altered Primary Forests (APF).

The study area is concentrated in one of the most extensive areas of bauxite deposits in the Amazon, which covers 50,000 km^2^ from eastern Pará to western Maranhão [64]. Bauxite exploration in the Amazon requires the removal of vegetation and soil from forested areas and follows a strip-mining model, where strips of land are mined and subsequently recovered. This soil can be excavated to a depth of 8 meters, and after removing the bauxite, the soil is in a sterile state [65]. To promote the forest’s natural recovery process, areas with suppressed vegetation undergo soil correction, recomposing the surface layers with inert material and relocating the forest’s surface layer (topsoil), previously removed from the forest during the suppression process [87–88]. After this process, the forest begins its recovery process naturally [63]. The Company applies other processes to accelerate forest recovery, such as nucleation and planting of native species; however, the areas that were the focus of this research only involved natural regeneration, which we called Recovering Forest Areas (RFA). All the Recovering Forest Areas (RFA) had their recovery processes started six to 11 years before the sampling period, including 2018, 2019 and 2020. In these areas of natural regeneration, the herbaceous/shrub component is dominated by families and species of early succession and colonizers, such as the genera *Cecropia*, *Anadenanthera*, *Annona*, *Byrsonima* and *Solanume* [63]. In a floristic study conducted in the same sampling areas of this study, only 17 shrub species were recorded across 9 plots of 1 ha each [63]. In the understory, the most common floristic species are *Vismia guianensis*, with high abundance, followed by *Croton matarrensis* and *Byrsonima crassifolia*, with lower occurrence in this stratum are the species *Solanum crinitum* and *Cecropia distachya* [63]. The canopy is discontinuous, with areas of exposed soil with little or no leaf litter. In the understory, the most common floristic species are *Vismia guianensis*, with high abundance, followed by *Croton matarrensis* and *Byrsonima crassifolia*, with lower occurrence in this stratum are the species *Solanum crinitum* and *Cecropia distachya* [63]. The canopy is discontinuous, with areas of exposed soil with little or no leaf litter. Therefore, these areas are considered in the early stages of recovery, as the first 20 years of succession are crucial to define differences in the floristic composition of landscapes [66].

### Sampling of small nonflying mammals

We conducted four field expeditions, each lasting eight to ten effective days of sampling, from November 2018 to March 2020, with two expeditions during the dry season (August and November) and two during the rainy season (January and March) [90]. We defined 12 sampling points, seven in APF and five in RFA, each at least 500 m apart (Fig 1). For spatialization, we followed the criterion of availability of areas with the same forest recovery treatment (natural regeneration) and restoration period, to avoid increasing the number of variables to be controlled in the study. At each sampling point, we installed a line of pitfall traps, consisting of a set of five buckets with a capacity of 60 liters, interconnected by an 80 cm high plastic fence with 10 cm buried in the ground [91] (S1 Fig). We buried the buckets up to the rim and spaced them 15 meters apart, forming an interconnected X-shaped set (Fig 1C).

We calculated the sampling effort by multiplying the total number of pitfall lines used (12) by the total number of effective sampling days (36). All 12 trap points were inspected daily throughout the collection period. We packed the captured animals in cloth bags and took them to the laboratory set up in the field to process the specimens. Due to the difficulty of identifying this group, some specimens were euthanized with an overdose of ketamine and xylazine, administering 0.1 mg of each substance for each 10 g of the animal’s mass [67]. The animal collection followed the guidelines of the American Society of Mammalogists [68] and was authorized by the Brazilian government through the permanent collection license SISBio 37174–1. The euthanized animals were taken to the Museu Paraense Emílio Goeldi (Belém, Pará, Brazil) and identified with the aid of cranial measurements and, in some cases, with karyotypic analysis. Easily identifiable animals were only sedated to record body measurements and identifiable features and then were released near the collection site on the same day (S1 Appendix).

### Specimen identification

The euthanized animals were prepared for taxonomic identification. Skulls were cleaned with multi-enzymatic detergent. The post cranial carcasses were stored in 70% ethanol. The skin, skulls, and teeth were used for taxonomic identification, as following: Faria and collaborators for Didelphimorphia [69], and Ferreira and collaborators for the genus *Marmosops* [70], and Patton and collaborators for the Rodentia [71], specifically the families Cricetidae and Echimyidae, and Saldanha and Rossi for the genus *Oecomys* [72]. The specimens are housed at Museu Paraense Emílio Goeldi (MPEG) (S1 Appendix).

### Collection of environmental variables

To verify the influence of environmental variables on the patterns of richness, composition and abundance of small nonflying mammals among the different habitats, we measured certain environmental variables at each pitfall collection point. The selected variables were those that reflect the habitat structure characteristics that can influence the parameters of small nonflying mammal communities [44]. These variables were used as covariates in the comparative analyzes between APF and RFA. The measured variables were: forest basal area, measured through Diameter at Breast Height (DBH), litter height, and percentage of land cover [45, 73–75].

Basal area is a measure of habitat structure related to vegetation biomass. We used two plots of 5 x 5 m for this variable, one on each side of the installed pitfall lines. Within the plots, all plants with circumference at breast height (CBH) above 10 cm were measured [76]. These measurements were transformed to DAP and the total basal area was calculated using the following formula = (DBH^2^ x π) / 4 [77], summing all values for each sampling unit.

We measured litter height by selecting four 0.25 m^2^ subplots at the corners of each basal area measurement plot. Using a 25 x 25 cm metal frame [45], we measured the litter height above the ground in millimeters, at the four corners of the subplot, with the aid of a ruler. In this way, we obtained 16 litter height measurements per sample plot and 32 measures per pitfall point. For analyses, we used the total average of these measurements [45] (S2 Fig).

To measure the percentage of land cover, we used mosaic of classified satellite images from 2019, with a 30-meter resolution, from collection seven of the MapBiomas platform, accessed through the following link (https://storage.googleapis.com/mapbiomas-public/brasil/collection-71/lclu/coverage/brasil_coverage_2019.tif). Using the QuantumGis 3.36 software (https://qgis.org/pt_BR/site/), we created test buffers with radius ranging from 100, 200, and 300 meters around each sample unit, based on the home range size of non-flying small mammals listed in the literature [78–81]. These buffers were used to quantify the percentage of land cover at the sampling points. By utilizing geoprocessing tools, we intersected the buffers with the classified and vectorized image from MapBiomas, to quantify each land use class. In this process, the classifications codes and description recorded within the buffers were: 3 (forest formation), 12 (grassland formation), 15 (pasture), and 30 (mining).

After extracting land use classes from MapBiomas, we refined these classifications through field validation and by using locally classified images to aid in this refinement. After validating the defined class categories for all sampling points, we recorded the following types of land cover: % of forest cover (% FC) and % of non-forest native vegetation cover (% NFC). With the categories defined, we tested which buffer size option (100, 200, or 300 meters) best represented the relationship with the total abundance of non-flying small mammal species at each sampling point. This test was conducted to select the best buffer size for subsequent analyses. To this end, we used a Canonical Correlation Analysis (CCA) and selected the 300-meter radius.

### Definition of functional traits

We followed the definition of Violle and collaborators, where a functional characteristic can be any morphological, physiological or phenological character measurable at the individual level [82]. This trait must be genetically or epigenetically heritable, and it needs to impact the species’ fitness, even if indirectly, through its effects on growth, reproduction and survival [82]. From this, we clarified that the functional characteristics must be related to the evaluated ecological process, in our case, the forest restoration. We selected four functional characteristics: habit, activity, trophic guild and body mass (S1 Table).

The type of activity reflects the temporal use of resources, and it is a definite trait classified as diurnal, crepuscular, nocturnal, and acyclic [83]. The habit is related to the use of space by species according to abiotic components such as soil depth, organic matter and water availability [84] and biotic features such as vegetation structural complexity [85], and it is also a definite trait classified as scansorial, arboreal and terrestrial. For the definition of both characteristics, we used databases and literature [69, 86, 87].

The trophic guild reflects the quality and quantity of resources consumed by the species, which is strongly related to the energy flow in the habitat [88]. To define trophic guilds, we used the classification by Wilman and collaborators and explained the following cut-off values: species with plant consumption equal to or greater than 50% were defined as granivore, species with fruit and seed consumption similar to or greater than 50% were described as frugivore, species with insect consumption equal to or greater than 50% was defined as insectivore and species with consumption less than 50% distributed among the various items was defined as an omnivore [95].

Body mass is related to the specific metabolic demand of the organism [89] and the risk of predation, acting strongly on the survival of the individual [54]. To calculate average body mass, it was first necessary to classify the individuals’ age. The age classification of rodents and marsupials relies on assessing molar wear and tooth eruption [90, 91]. Juvenile status is assigned when there’s incomplete eruption of molars and premolars (in marsupials), or when molar wear is absent [90, 91]. Conversely, adulthood is recognized by the complete eruption of all teeth, including molars and premolars, accompanied by visible molar wear at various levels [90, 91]. A mean body mass was calculated when 2 to 5 adult individuals were available. For species with fewer than two adult individuals, we used the body mass from the literature [92]. We excluded specimens identified only at the genus level from the analyses, such as *Oecomys* sp. (S2 Table).

### Data analysis

#### Evaluation of the taxonomic structure

We used rarefaction and extrapolation curves based on Hill numbers [93], incorporating the abundance of species as response variable. This analysis estimates taxonomic diversity considering sample size and coverage (predictor variable) [93]. It calculates three different metrics based on the q parameter, which determines the sensitivity of the measurement concerning the relative frequencies of the species, generating rarefaction and extrapolation curves for each. Considering the three metrics, in the first one, we evaluated species richness, where the value of q = 0, and abundance does not contribute to the sum of the equation, generating a rarefaction and extrapolation curve for sample coverage. In the second metric, we evaluated Shannon diversity (q value = 1), in which species are weighted according to their frequencies. Third, we generated a rarefaction and extrapolation curve for Simpson’s diversity (q value = 2), in which species with more abundant frequencies are given greater weight and rarer species are disregarded [93].

We elaborated the accumulation curves from rarefaction and extrapolation, where the samples were standardized by coverage levels and equal size, in addition to integrating both rarefaction and extrapolation of data, which is useful to estimate absolute values of diversity in small samples and between environments with unequal sampling [94]. The analyses were performed in the R Program, in the iNEXT package, using the following functions: iNEXT, ggiNEXT and estimateD [93, 95].

We performed a Multidimensional Scaling Analysis (NMDS) to graphically verify the dissimilarity of species composition between samples from each habitat category studied, using the Bray-Curtis Similarity Index [96]. We used the MASS package and the isoMDS function to performed this analysis. We then performed a Multivariate Permutation Analysis of Variance (PERMANOVA) to statistically test the difference of the composition of species between APF and RFA samples. We also used a dispersion homogeneity test (PERMDISP) to statistically analyze whether samples from each habitat were heterogeneous [97]. We used the vegan package and the adonis2 and betadisper functions [98].

#### Evaluation of the influence of environmental variables

Initially, we selected the 300-meter buffers to extract the land use variables, as it was the buffer size that proved to be the most appropriate considering the structure of the small mammal community. Subsequently, we performed a VIF Correlation Analysis to remove correlated variables among the selected ones: % FC, % NFC, litter, and basal area [97]. We used the car package and the vif function for this analysis. To evaluate the spatial effect of the samples and the environment separately on the composition of the small mammal community, we performed a Partial Redundancy Analysis (RDAp). This analysis partitions the impact of the predictor variables on the dependent variable [98]. To relate and graphically visualize the effects of environmental variables on small mammals’ species composition, we used a Canonical Correspondence Analysis (CCA). This analysis orders the environmental data matrix, relating it to the community composition through multiple regressions, excluding spatial dependence [99]. We used the vegan package and the CCA and RDA functions for both analyses [96].

#### Evaluation of the functional structure

To assess functional distances between species, we employed Pavoine’s mixed-variable distance coefficient [99]. This coefficient accommodates a mix of different statistical types such as quantitative, binomial, and fuzzy variables, allowing us to generate a cluster dendrogram for each sampled habitat using generalized Gower distance. We applied a distance cutoff of 0.5 to identify the formation of functional groups and to measure the functional distances between species [99]. This approach enabled us to more accurately delineate functional groups within each habitat. To evaluate the functional traits of small mammal communities across habitats, we utilized several metrics such as Functional Richness (FRic), which represents the volume of functional space (niche) occupied by all species within a community [100]; Functional Evenness (FEve), which calculates the sum of branch lengths in the multidimensional trait space, weighted by species’ relative abundance [100] and Functional Divergence (FDiv), which measures the deviation of species from the mean distance to the center of gravity, also weighted by relative abundance [100]. All functional metrics were computed using R (version 3.5.3) with the "FD" package and the "dbFD" function, based on a functional distance matrix derived from Pavoine’s mixed-variable distance and a matrix of species abundance and community composition [101]. Each calculated index was evaluated individually, between the areas of APF and RFA, using a student’s t test located in the car and RVAideMemoire packages and the byf.shapiro, leveneTest and t.test functions [102].

To observe how the functional characteristics were related to the sampled habitats, with the environmental variables measured and with the species, we performed an RLQ ordering, which is an extension of a Co-Inertia analysis, where three data matrices are jointly treated and allow the visualization between the relationship of these matrices [103]. The analysis calls the set of environmental variables by sampling units the R table, while the L table is the one for the presence and absence of species by sampling unit, and the Q table consists of the set of functional characteristics found in the species present in the sampling units [103]. To assess the significance of the associations between the analysis matrices we used two permutation models combining the RLQ and fourth-corner approaches [104]. Model 2 evaluates the relationship between the matrix of environmental variables and the species occurrence matrix, while Model 4 examines the association between the matrix of species functional traits and the matrix of environmental variables [105]. The matrix R and Q were analysed by Hill-Smith Principal Component Analysis (PCA), and matrix R and L by Correspondence Analysis (CA) ordination [104]. We used the vegan package and the rlq function [98]. All analyses developed in the study were calculated using the R program version 3.5.3. [106], with the significance value of the tests considered as P < 0.05.

## Results

### Evaluating the taxonomic structure

We employed a sample effort of around 432 traps/night, 252 traps/night in the APF areas and 180 traps/night in the RFAs. This resulted in the capture of 105 individuals, 74 rodents and 31 marsupials. In total, we recorded 23 species of small nonflying mammals, 16 of which were rodents (four Echimyidae and 12 Cricetidae) and seven marsupials. In the RFAs, we recorded 43 individuals of 10 species, and in the APF areas, we recorded 62 individuals of 22 species (S3 Table).

**Fig 2:**
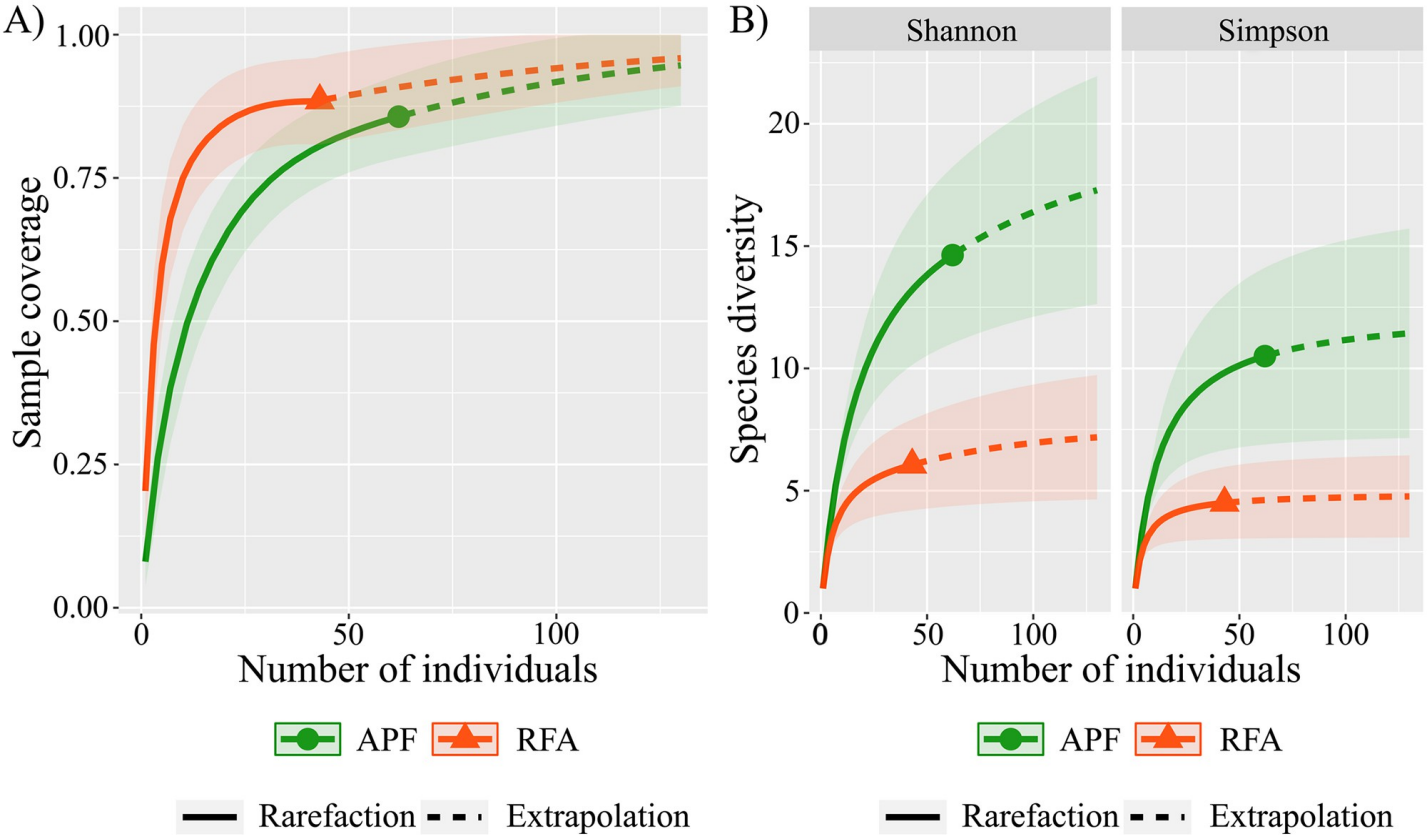
Graphical representation of the rarefaction and extrapolation curve for small nonflying mammal species, with 95% confidence intervals. The green lines represent the Altered Primary Forest (APF), and the orange lines represent Forest Recovery Areas (RFA). (A) Shows the sample coverage (q = 0) in each studied habitat (B) Shows the curves for Shannon diversity (q = 1) considering species abundance, and Simpson diversity (q = 2) considering more frequent species and disregarding rarer species.

We found no differences in the taxonomic richness of small non-flying mammal species between the RFA and APF habitats (Fig 2A). However, the rarefaction and extrapolation curves for sample coverage (Fig 2A) showed that for RFA the maximum species richness found was reached with fewer individuals collected than in APF, and the rarefaction curve stabilizes faster in RFA, due to the lower species richness. However, we found differences between the RFA and APF habitats in terms of Shannon and Simpson diversity (Fig 2B). It is worth remembering that Shannon diversity considers the abundance of individuals per species, while Simpson diversity gives greater weight to more frequent species and disregards rare ones. In both cases, APF presented greater diversity than RFA, since there was no overlap between the curves (Fig 2B). The RFA habitat presented less equitability in species abundance, with a few more abundant species and the remainder of rare species (S3 Table).

**Fig 3:**
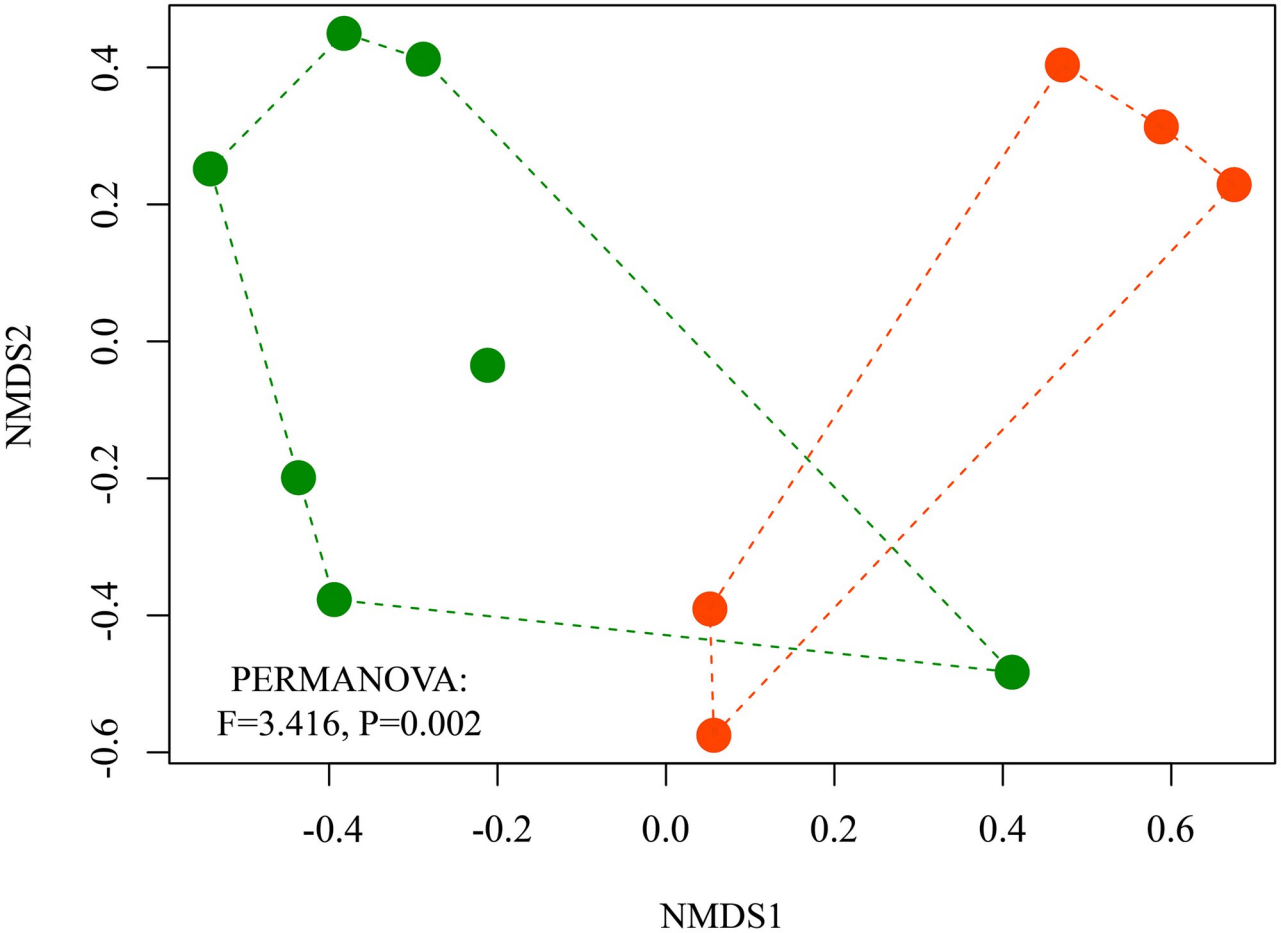
Dissimilarity in species composition of small nonflying mammals. Results of the Multidimensional Scaling Analysis (NMDS) and the PERMANOVA, comparing the composition between samples from Altered Primary Forest habitats (in green) and Forest Recovery after bauxite mining (in orange).

**Fig 4:**
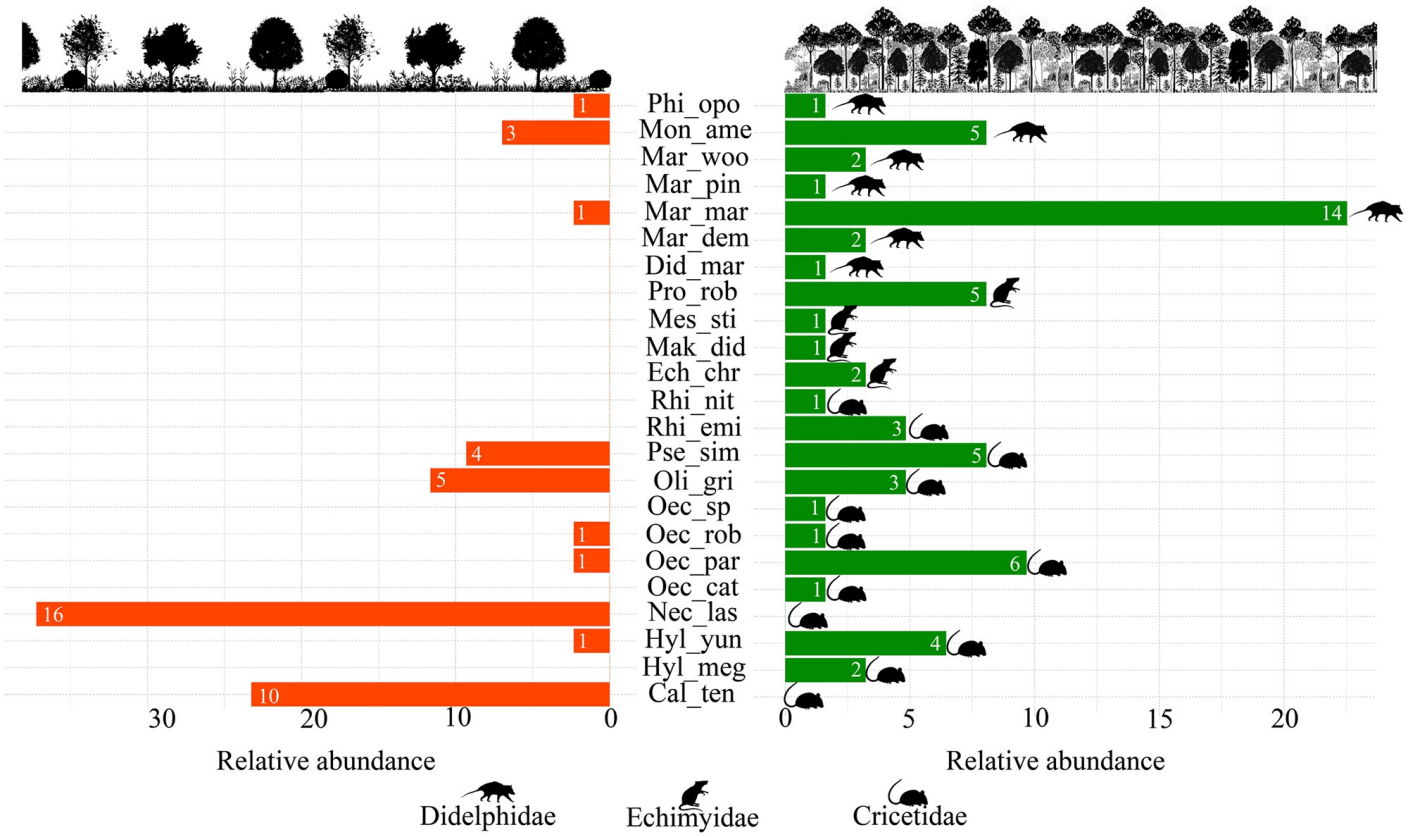
Composition and relative abundance of species of small nonflying mammals. Areas of altered primary forest are represented by green bars and areas of forest recovery after bauxite mining by orange bars. Relative abundance represents the percentage of records for each species in each habitat. The upper part of the graph shows rodent species, and the lower part represents marsupials. Legend of the species: (Cal_ten) *Calomys tener*, (Did_mar) *Didelphis marsupialis*, (Ech_chr) *Echimys chrysurus*, (Hyl_ yun) *Hylaeamys yunganus*, (Hyl_meg) *Hylaeamys megacephalus*, (Mar_dem) *Marmosa demerarae*, (Mar_mar) *Marmosops marina*, (Mar_pin) *Marmosops pinheiroi*, (Mar_woo) *Marmosops woodalli*, (Mak_did) *Makalata didelphoides*, (Mes_sti) *Mesomys stimulax*, (Mon_ame) *Monodelphis americana*, (Nec_las) *Necromys lasiurus*, (Oec_rob) *Oecomys* cf. *roberti*, (Oec_cat) *Oecomys* gr. *catherinae*, (Oec_par) *Oecomys* gr. *paricola*, (Oec_sp) *Oecomys* sp., (Oli_gri) *Oligoryzomys gri apinaye*, (Phi_opo) *Philander opossum*, (Pro_rob) *Proechimys roberti*, (Pse_sim) *Pseudoryzomys simplex*, (Rhi_emi) *Rhipidomys emiliae*, (Rhi_nit) *Rhipidomys nitela*.

In addition to the difference in diversity, we also observed variation in species composition between samples from the APF and RFA habitats (PERMANOVA: F = 3.416, P = 0.002) (Fig 3). Eight of the 23 species recorded were common to both habitats (Fig 4). In RFA, we recorded two exclusive and abundant species: *Calomys tener* (Winge, 1887) and *Necromys lasiurus* (Lund, 1840). While in the APF, we recorded 13 exclusive species, being the marsupials, *Didelphis*. *marsupialis*, *Marmosa demerarae* Thomas, 1905, *Marmosops pinheiroi* and *Marmosops woodalli* (Pine, 1981), and the rodents *Echimys chrysurus*, *Hylaeamys megacephalus* (G. Fischer, 1814), *M*. *didelphoides*, *M*. *stimulax*, *O*. *roberti*, *O*. *catherinae*, *P*. *roberti*, *Rhipidomys emiliae* (J. A. Allen, 1916) and *R*. *nitela*. The most common species were *N*. *lasiurus* and *Marmosops marina* Ferreira 2020 (Fig 4).

**Fig 5:**
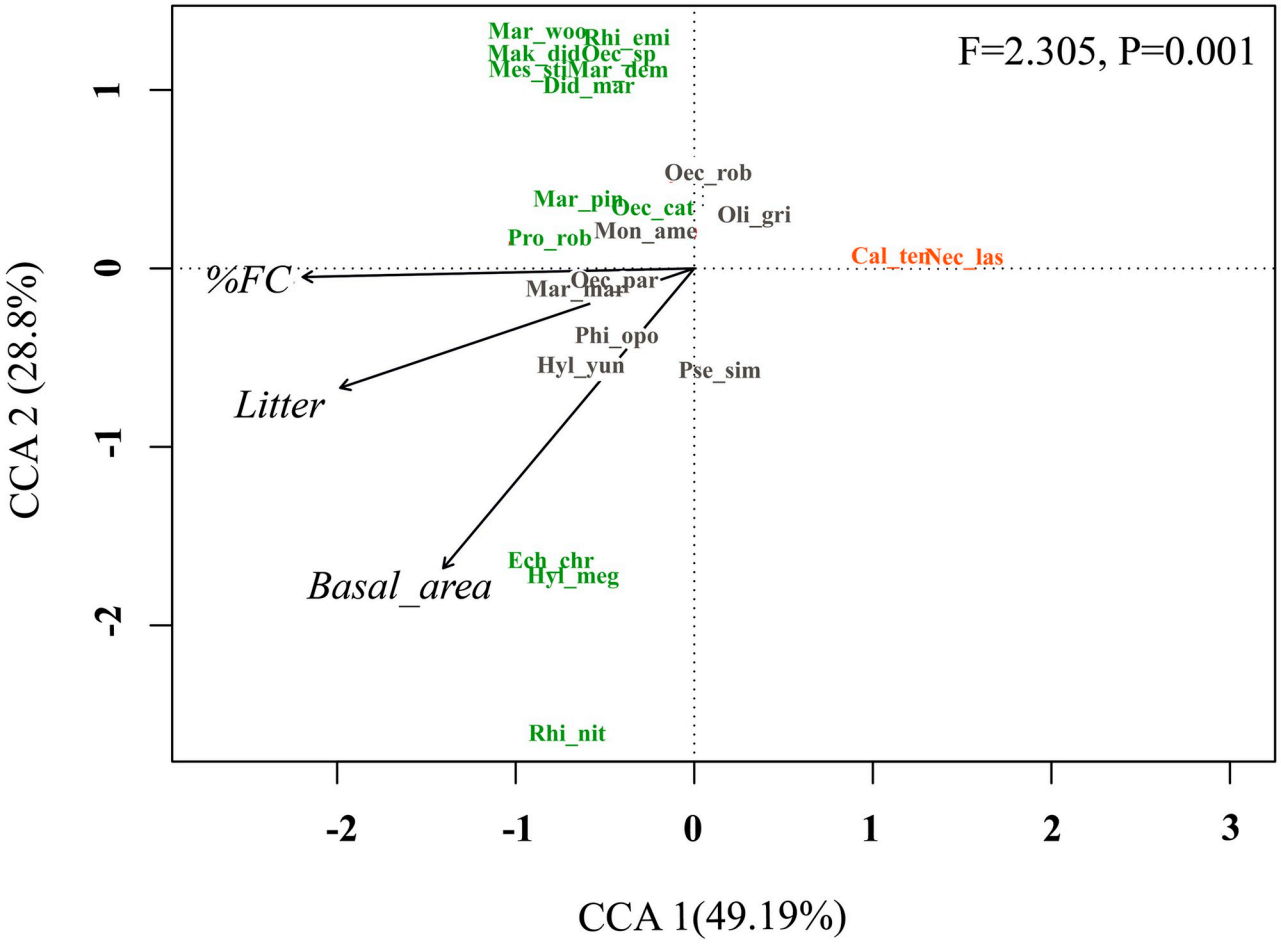
Result of Canonical Correspondence Analysis (CCA) evaluating the influence of environmental variables on the species composition of small non-flying mammals. Legend to environmental variables: (% FC) Percentage of forest cover, (Litter) Litter quantity in mm, (Basal_area) Basal area quantity in square meters. Legend for the species: (Cal_ten) *Calomys tener*, (Did_mar) *Didelphis marsupialis*, (Ech_chr) *Echimys chrysurus*, (Hyl_ yun) *Hylaeamys yunganus*, (Hyl_meg) *Hylaeamys megacephalus*, (Mar_dem) *Marmosa demerarae*, (Mar_mar) *Marmosops marina*, (Mar_pin) *Marmosops pinheiroi*, (Mar_woo) *Marmosops woodalli*, (Mak_did) *Makalata didelphoides*, (Mes_sti) *Mesomys stimulax*, (Mon_ame) *Monodelphis americana*, (Nec_las) *Necromys lasiurus*, (Oec_rob) *Oecomys* cf. *roberti*, (Oec_cat) *Oecomys* gr. *catherinae*, (Oec_par) *Oecomys* gr. *paricola*, (Oec_sp) *Oecomys* sp., (Oli_gri) *Oligoryzomys gri apinaye*, (Phi_opo) *Philander opossum*, (Pro_rob) *Proechimys roberti*, (Pse_sim) *Pseudoryzomys simplex*, (Rhi_emi) *Rhipidomys emiliae*, (Rhi_nit) *Rhipidomys nitela*.

Considering the VIF correlation results, we removed the % NFC variable from the analyses. The spatial influence analysis (RDAp) showed that the environmental differences of the habitats influenced more than the spatial distribution of the samples. Environmental variables explained 19% of the variation in the studied assemblage (F = 1.92, P = 0.01), while spatial distribution explained only 9% of the variation found in the community (F = 1.59, P = 0.07) (S4 Table). The Canonical Correspondence Analysis (CCA) explained 77.99% of the data variation in the first two axes, 49.19% in the first, and 28.8% in the second axes (S5 Table). The variables that most contributed to the formation of the axes of the APF samples were the more significant amount of litter and % FC. The rodents *Rhipidomys nitela*, *Hylaeamys megacephalus* and *Echimys chrysurus* were strongly influenced by lower values of basal area; the opossum *Philander opossum* (Linnaeus, 1758) and the rodent *Hylaeamys yunganus* (Thomas, 1902) were influenced by the higher values of variables litter and basal area; and the opossum *Marmosops marina* and the rodent *Oecomys* gr. *paricola* (Thomas, 1904) were affected by the higher values of variables % FC and litter. *Necromys lasiurus* and *Calomys tener*, found exclusively in the RFA, were influenced by environmental characteristics of more open areas with low forest cover percentage, low litter levels and basal area (Fig 5).

### Evaluating the functional structure

**Fig 6:**
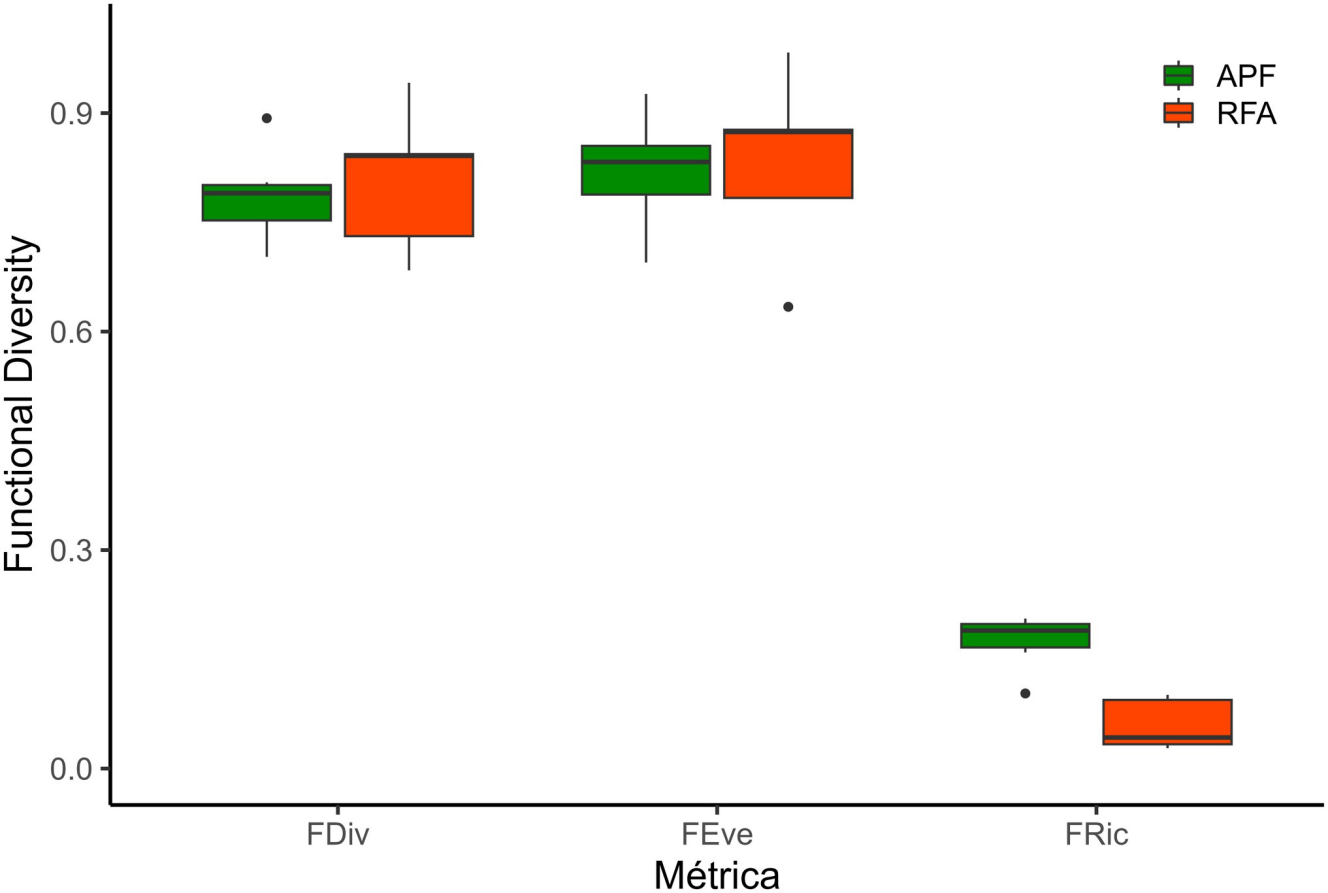
Results of the functional diversity analyses of small non-flying mammals, considering the three metrics analyzed. (FDiv) Functional Divergence; (FEve) Functional Eveness; and (FRic) Functional Richness. The boxplots in green represent Altered Primary Forest (APF) values and Forest Recovery Area (RFA) in orange.

We found that functional divergence (FDiv) and functional evenness (FEve) were not significantly different between the habitats (T = 0,50, GL = 10, P = 0,8 and T = -0,17, GL = 10, P = 0,86, respectively) (Fig 6). However, the number of functional spaces occupied, characterizing the functional richness (FRic) of small non-flying mammals in the APF, was significantly higher than in the RFA (T = 5,56, GL = 10, P = 0,0002) (Fig 6).

**Fig 7:**
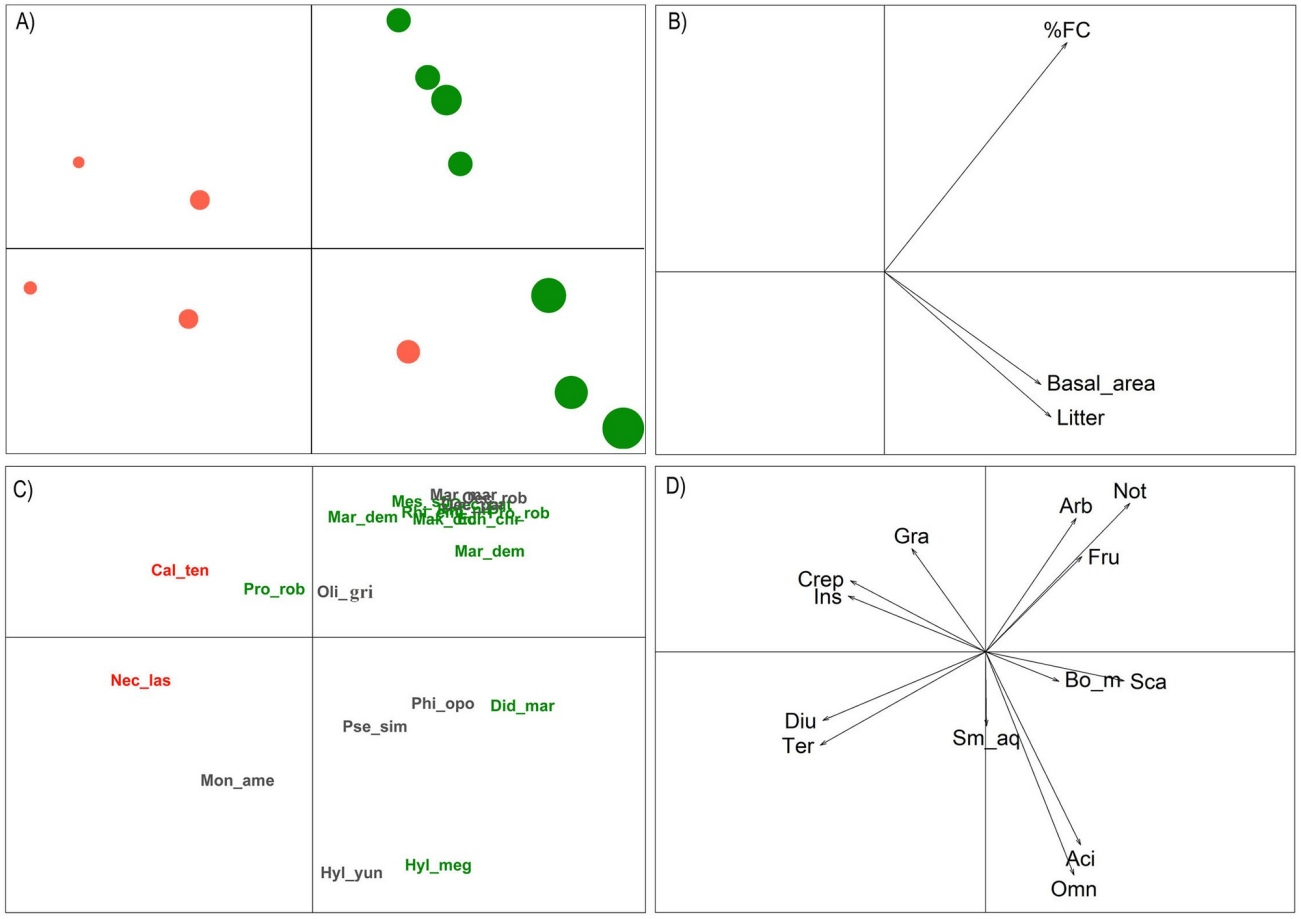
Results of the RLQ analysis show the relationship of functional traits of small non-flying mammal species concerning environmental variables. (RFA) Areas of Forest Recovery after bauxite mining, represented by the axes on the left, in orange, and (APF) Areas of Altered Primary Forest, defined by the axes on the right, in green. Legend of types of habits: (Arb) arboreal, (Sca) scansorial, (Sm_aq) semi-aquatic, and (Ter) terrestrial; Period of activity: (Aci) No specific pattern, (Crep) Crepuscular, (Diu) diurnal, (Noc) Nocturnal; Diet: (Fru) Fruitivore, (Gra) Granivore, (Ins) Insectivore, and (Omn) Omnivore; and (Bo_m) Body mass.

Overall, we did not find a significant relationship between functional characteristics of the small mammal community and environmental variables through RLQ ordination analysis (Model 4, P = 0.095). However, some specific functional aspects, such as higher body mass index, are correlated with higher basal area, litter, and % FC indices (Fig 7). Furthermore, species with greater body mass are associated with frugivorous and omnivorous diets and the predominance of arboreal and scansorial habits. This entire set of characteristics is related to the APF areas (Fig 7). On the other hand, the lowest values of basal area, litter, and % FC are related to the RFAs, where terrestrial habits predominate, as well as species with crepuscular activity and granivorous and insectivorous diets (Fig 7).

## Discussion

Our study shows that in the early stages of forest restoration (6–11 years) after bauxite mining, it is possible to recover just over to 45% of the richness of small nonflying mammals compared to forest habitats similar to the original ones, which in this case are the Altered Primary Forests. This percentage of species richness restoration. However, this percentage of recovered richness does not characterize the recovery of taxonomic or functional diversity at this stage of forest succession. Although 34.8% of the species composition is shared between the APFs and RFAs, the abundance distribution per species is less uniform in the RFAs. This inequitability in abundance among species reduces the taxonomic diversity indices and influences a functional diversity in the recovering forests.

In general, the recovery processes of vertebrate fauna after anthropogenic disturbances in tropical regions follow a gradual increase in species richness and restructuring of composition throughout the forest succession process [41, 107]. It is important to remember that our results showed responses from the initial stages of forest succession. More advanced phases, in intermediate and late stages, may show patterns of differentiated mammal communities, and the most significant difference may be in the abundance of some species, which may increase with the more complex structuring of the vegetation as the forest recovers [41]. Over time, new patterns may alter the Shannon and Simpson diversity, which in the initial stages of recovery differed significantly between the habitats compared. These diversity indices consider the frequency and abundance of species, excluding the effect of rare species in the case of Simpson [91]. In the medium and long term, the increase in the abundance of some species may positively influence the diversity of the areas undergoing recovery [41].

However, other variables may influence the future responses of these animals to forest recovery, including the type of impact suffered, the surrounding landscape, the remaining biological legacy, and environmental filters [23–26]. In our study, it is essential to highlight that the original habitat before bauxite mining was composed of an Altered Primary Forest; the result could also be different if the original forests were preserved. Some studies using an integrated approach to assess impacts on mammal groups have also observed that taxonomic diversity patterns recovered faster than species composition [49, 50, 108]. Composition and diversity may influence the functionality of this taxonomic group in the impacted ecosystem. In our study, the RFA is probably still an extreme environment, acting as an environmental filter in the community of small non-flying mammals, within which species with similar functions are selected due to their adaptations to the environment and ability to colonize it, thus functioning as pioneer species.

One of the main objectives of restoring altered habitats is the recovery of ecosystem functions [51, 107]. It is often assumed that increasing species or functional richness is equivalent to restoring ecosystem function. However, as demonstrated in our work, this is not always the case. Restoration of species richness did not directly influence functional richness, which differed between APF and RFA assemblages. This result shows that APF has a more significant number of ecological functions performed by small non-flying mammals than in RFA. At the same time, RFA mainly comprises a few abundant pioneer species with similar functional traits, leading to lower values of functional richness. However, the metrics influenced by abundance, such as Functional Divergence and Functional Evenness did not differ between the habitats.

Nevertheless, the functional divergence values were high for both habitats, which suggests that in both APF and RFA the species present high functional divergence and are distributed far from the center of the functional space, which may allow the coexistence of distinct species, each occupying specific niches in the multidimensional space [109]. Furthermore, the high values of Functional Uniformity for both habitats suggest that in both APF and RFA the abundance of species is evenly distributed throughout the functional space [109]. Thus, the structure of the assemblages of small mammals in initial habitats of forest recovery after bauxite mining would be less complex than the assemblages of APF, presenting a smaller number of ecological functions, but presenting uniformity among them.

Concerning species composition, we highlight the exclusive presence of *N*. *lasiurus* and *C*. *tener* in RFA, related to the lowest values of % FC, litter and basal area. These species mainly inhabit open areas of Cerrado and Caatinga, in addition to monocultures and pastures [71], and are probably favored by recovery areas in a scenario of a positive effect of open forest on the abundance of terrestrial species [42]. These two species have the most abundant and active functional characteristics in this environment, where the predominant diet is granivorous and insectivorous, the habit is terrestrial, and the activity is diurnal and crepuscular [87]. Based on these data and the result of the FDiv metric for RFA, we observed that there are high rates of dispersion of functional traits, which may be a reflection of a high level of competition between species with similar characteristics and the absence of ecological solid filters in this community [110], also contributing to lower species diversity.

Notably, the registration of the species *C*. *tener* was the first in the state of Pará, expanding its geographic distribution. The closest record of this species available in the literature occurs in the state of Tocantins [111], suggesting that its distribution is probably underestimated due to a lack of sampling, or this species may be favored by environmental degradation in the Amazon, since the location of the studied area is framed in the region of the arc of Amazon deforestation [61], close to transition regions between the Amazon and Cerrado biomes in the state of Tocantins.

We note that *Monodelphis americana* (Müller, 1776), *Oligoryzomys gri apinaye* (Bonvicino & Weksler 2024) and *Pseudoryzomys simplex* (Winge, 1887), were species with high abundances in both habitats. Despite being a forest species, the marsupial *M*. *americana* is tolerant to several types of habitats [112], whereas the rodents *O*. *gri apinaye* and *P*. *simplex* occupy forest formations, such as semi-deciduous and riparian forests, in addition to transition areas with edge effect [71]. These species share common functional characteristics in the RFA areas, such as terrestrial habits, and predominant features of forest areas, such as frugivorous and omnivorous diet and semi-aquatic habit.

Based on this distribution of functional traits and the result of the functional richness, we observe that there probably that there are still unique ecological attributes as new species are added in RFA [113]. In the medium and long term, we expect that the greater probability of the presence of new traits will reflect in the reduction of functional redundancy and increase the resilience to environmental disturbances of the assemblage [114]. Expanding food niches can also reduce intraspecific competition [115]. It is essential to highlight that all these species can actively contribute to forest restoration, as they can transport seeds and seedlings from forest areas to areas undergoing restoration [16].

Our results showed that several species are restricted to APF areas. These species are related to higher rates of FC %, litter and basal area, with typical functional characteristics of forest areas, such as nocturnal and acyclic activity, arboreal and scansorial habits, frugivorous and omnivorous diet and high average body mass [87, 92]. The positive relationship between leaf biomass and abundance of small mammal species with arboreal and scansorial habits was also observed in the Atlantic Forest [116]. The recompositing of the forest structure over time, allowing for greater complexity of the understory, greater canopy density, and an increase in litter and fallen trunks, enables an increase in the abundance and richness of species while restoring the structure of species composition in a small mammal community [42]. A more complex habitat represents greater vertical heterogeneity and greater availability of shelter and food [117], which can change the relationships observed between species and environmental variables, in addition to the availability of niches and ecological functions. Thus, the relationships and parameters found in the initial phases of the intermediate stage of forest recovery in the present study may change over time.

Even if the RFA has not fully recovered the functions performed by small nonflying mammals in APF, the recovered functionality is essential in the intermediate stages of succession. We highlight the “granivore” trophic guild category, which is extremely necessary for energy flow, nutrient cycling and plant dynamics in the intermediate stages of forest recovery [118]. This ecological function allows the suppression or limitation of seedling growth of pioneer species, allowing the regeneration of plant species of later successional stages [16]. In addition, we also observed the predominance of the insectivorous diet, which controls the abundance of invertebrates, which tend to be very abundant in the intermediate stages of succession. This control directly reflects on plant composition [119]. All of these functions end up altering the structural and floristic results in the forest restoration process, favoring and accelerating ecological succession, especially in the intermediate stages [67, 68, 117].

We can conclude from our results that in the early stages of forest succession (between 6 and 11 years) after bauxite mining, it is still unable to recover the taxonomic or functional of the fauna of small non-flying mammals in the Amazon. However, it is possible to recover functional divergence and functional evenness, although functional richness has not been reestablished. Species such as *N*. *lasiurus* and *C*. *tener* can be highlighted as indicators of areas in the early stages of recovery in the study area, not only because of their exclusivity in this habitat but also because of their high abundance and relationship with the characteristics of open areas, such as low litter density and basal area. On the other hand, species such as *M*. *marina* and *O*. *paricola* can be highlighted as indicators of forest areas, considering the relationship of these species with the increase in the percentage of forest.

In addition to providing important information for monitoring the restoration of degraded areas, our results show that the approach of different metrics of functional diversity, along with taxonomic diversity, allows for more detailed assessments of the forest restoration process [120]. However, the monitoring small mammals over time would be important to understand the response of this fauna to the recovery process in intermediate and late successional scenarios. Thereby, we reinforce the need to monitor and maintain long-term recovery programs, in addition to investigate the responses of other biological groups.

## Supporting information

S1 Appendix Details of registered specimens. (DOCX)

S1 Fig Photos of sampling points. (A) Pitfall Traps installed in Forest Recovery Areas (FRA) and (B) in Altered Primary Forest (APF) areas.
(TIFF)

S2 Fig Layout of plots and sub-plots delimited for collecting environmental variables. (A) Basal area sampling, in 5 x 5 m plots; (B) Litter height sampling in 25 x 25 cm subplots.
(TIFF)

S1 Table Description of functional traits used for functional analyses, including their measurement units, value categories, and functional meanings. (DOCX)

S2 Table Morphological and functional attributes of the species of rodents and marsupials sampled. Habit (locomotion): (Sc) Scansorial, (Ar) Arboreal, (Te) Terrestrial, (Saq) Semi-aquatic; Period of activity: (Noc) Nocturnal, (Cre) Crepuscular, (Day) Daytime, Aci (Acyclic); Trophic guild/diet: (Inv) Invertebrates; (Vend) Mammals and birds; (Vect) Reptiles and amphibians; (Vunk) Vertebrates in general or unidentified (Scav) Organic garbage and carrion, (Fru) Fruit, (Nec) Nectar and pollen, (See) Seeds, spores and grains, (Pla) Grass, seedlings, tubers, bulbs, lichens, moss, roots, twigs, bark and leaves, (Fun) Mycorrhizal fungi; Morphology: (Bod_m) Body mass (in grams).
(DOCX)

S3 Table The taxonomic structure (composition, richness and abundance) recorded in the small mammal community in the studied areas. Abundance (N) Number of specimens sampled in RFA (Recovery Forest Area) and APF (Altered Primary Forest).
(DOCX)

S4 Table Result of the Partial Redundancy Analysis, evaluating the influence of the environment and space in the community. (DOCX)

S5 Table Percentage of explanation of the variables for each axis of the Canonical Correlation Analysis. (DOCX)
