# Supplementary material for: Multiparameter analysis of small non-flying mammals’ response to forest restoration post-bauxite mining in eastern Amazonia
Source: PLoS One. 2025 Jan 24;20(1):e0315904. doi: 10.1371/journal.pone.0315904 (PMC11759357; doi:10.1371/journal.pone.0315904)
Supplement: S1 Appendix — (DOCX) [file pone.0315904.s001.docx]

**S1_Appendix.** Details of collected specimens

The 98 specimens collected are held in the following collection: MPEG (Museu Paraense Emílio Goeldi). The localities of specimens are listed according to the geographic samples shown in fig 1. Numbers in square brackets refer to the localities show in fig.1. Underlined numbers refer to specimens without a skull.

Brazil: Pará state, Paragominas, Hydro Company:

[1] Forest IA, Miltonia 5 (3° 15.123'S, 47° 49.113'O): *Didelphis marsupialis* (MPEG 46218); *Marmosops marina* (MPEG 46193, MPEG 46208); *Marmosops woodalli* (MPEG 46130); *Monodelphis americana* (MPEG 46124, MPEG 46182); *Oecomys* gr. *paricola* (MPEG 46184); *Oecomys* cf. *roberti* (MPEG 46126); *Oligoryzomys gri apinaye* (MPEG 46140, MPEG 43153); *Proechimys roberti* (MPEG 46135, MPEG 46210); *Rhipidomys emiliae* (MPEG 46156, MPEG 46158).

[2] Forest IB, Miltonia 5 (3° 15.069'S, 47° 49.024'O): *Hylaeamys yunganus* (MPEG 46179); *Makalata didelphoides* (MPEG 46204); *Marmosa demerarae* (MPEG 46205), *Marmosops marina* (MPEG 46203, MPEG 46209); *Marmosops woodalli* (MPEG 46136); *Oecomys* gr. *paricola* (MPEG 46148); *Oecomys* sp (MPEG 46178); *Pseudoryzomys simplex* (MPEG 46199), *Rhipidomys emiliae* (MPEG 46149).

[3] Forest IIA, Miltonia 5 (3° 15.399'S, 47° 48.389'O): *Hylaeamys megacephalus* (MPEG 46155); *Hylaeamys yunganus* (MPEG 46177); *Marmosops marina* (MPEG 46157, MPEG 46160, MPEG 46162, MPEG 46163, MPEG 46180); *Oecomys* gr. *paricola* (MPEG 46133, MPEG 46183); *Proechimys roberti* (MPEG 46132, MPEG 46134); *Pseudoryzomys simplex* (MPEG 46138, MPEG 46145).

[4] Forest IIB, Miltonia 5 (3° 15.406'S, 47° 48.357'O): *Echimys chrysurus* (MPEG 46213); *Hylaeamys megacephalus* (MPEG 46154); *Hylaeamys yunganus* (MPEG 46211); *Marmosops marina* (MPEG 46142); *Oecomys* gr. *paricola* (MPEG 46141); *Pseudoryzomys simplex* (MPEG 46146, MPEG 46150), *Rhipidomys nitela* (MPEG 46143).

[5] Forest IIIA, Mineroduto road (3° 13.460'S, 47° 45.197'O): *Marmosops marina* (MPEG 46219); *Marmosops pinheiroi* (MPEG 46165); *Oecomys* gr. *catherinae* (MPEG 46151); *Proechimys roberti* (MPEG 46181).

[6] Forest IIIB, Mineroduto road (3° 13.407'S, 47° 45.227'O): *Marmosops marina* (MPEG 46220); *Mesomys stimulax* (MPEG 46174); *Oecomys* gr. *paricola* (MPEG 46215).

[7] Forest IV, Mining road (3° 14.262'S, 47° 43.983'O): *Echimys chrysurus* (MPEG 46129); *Hylaeamys yunganus* (MPEG 46175); *Marmosops marina* (MPEG 46128, MPEG 46217); *Monodelphis americana* (MPEG 46170).

[8] PRAD I, Cabeça de Anta, Natural Regeneration 2009 (3° 15.677'S, 47° 42.889'O): *Hylaeamys yunganus* (MPEG 46159); *Monodelphis americana* (MPEG 46168); *Oligoryzomys gri apinaye* (MPEG 46207).

[9] PRAD II, Jurubeba, Nucleation 2014 (3° 15.765'S, 47° 41.902'O): *Calomys tener* (MPEG MPEG 46123, MPEG 46127, MPEG 46172, MPEG 46173, MPEG 46198, MPEG 46202, MPEG 46206); *Monodelphis americana* (MPEG 46131); *Necromys lasiurus* (MPEG 46137, MPEG 46139, MPEG 46144, MPEG 46161, MPEG 46166, MPEG 46167, MPEG 46197, MPEG 46214); *Oecomys* cf. *roberti* (MPEG 46164); *Oligoryzomys gri apinaye* (MPEG 46169); *Pseudoryzomys simplex* (MPEG 46125, MPEG 46192).

[10] PRAD III, Natural Regeneration 2014 (3° 15.412'S, 47° 42.167'O): *Calomys tener* (MPEG 46171, MPEG 46200); *Necromys lasiurus* (MPEG 46152); *Oligoryzomys gri apinaye* (MPEG 46212), *Pseudoryzomys simplex* (MPEG 46194).

[11] PRAD IV, Muruci, Natural Regeneration 2014 (3° 14.945'S, 47° 42.342'O): *Calomys tener* (MPEG 46191); *Necromys lasiurus* (MPEG 46185-46189, MPEG 46195, MPEG 46201); *Oecomys* gr. *paricola* (MPEG 46176); *Oligoryzomys gri apinaye* (MPEG 46196); *Pseudoryzomys simplex* (MPEG 46190).

[12] PRAD V, Nucleation 2013 (3° 14.536'S, 47° 42.085'O): *Marmosops marina* (MPEG 46147); *Oligoryzomys gri apinaye* (MPEG 46216).

The seven specimens recorded in the present study and which were not collected. The localities of specimens are listed according to the geographic samples shown in Fig 1. Numbers in square brackets refer to the localities show in fig.1.

Monodelphis americana: [6] Forest IIIB, Mineroduto road (3° 13.407'S, 47° 45.227'O); [7] Forest IV, Mining Road (3° 14.262'S, 47° 43.983'O); e [12] PRAD V, Nucleation 2013 (3° 14.536'S, 47° 42.085'O).

Marmosa demerarae: [1] Forest IA, Miltonia 5 (3° 15.123'S, 47° 49.113'O).

Philander opossum: [3] Forest IIA, Miltonia 5 (3° 15.399'S, 47° 48.389'O); [8] PRAD I, Cabeça de Anta, Natural Regeneration 2009 (3° 15.677'S, 47° 42.889'O).

Oligoryzomys gri apinaye: [5] Forest IIIA, Mineroduto road (3° 13.460'S, 47° 45.197'O).
