# Supplementary figures and images for: Multiparameter analysis of small non-flying mammals’ response to forest restoration post-bauxite mining in eastern Amazonia
Source: PLoS One. 2025 Jan 24;20(1):e0315904. doi: 10.1371/journal.pone.0315904 (PMC11759357; doi:10.1371/journal.pone.0315904)

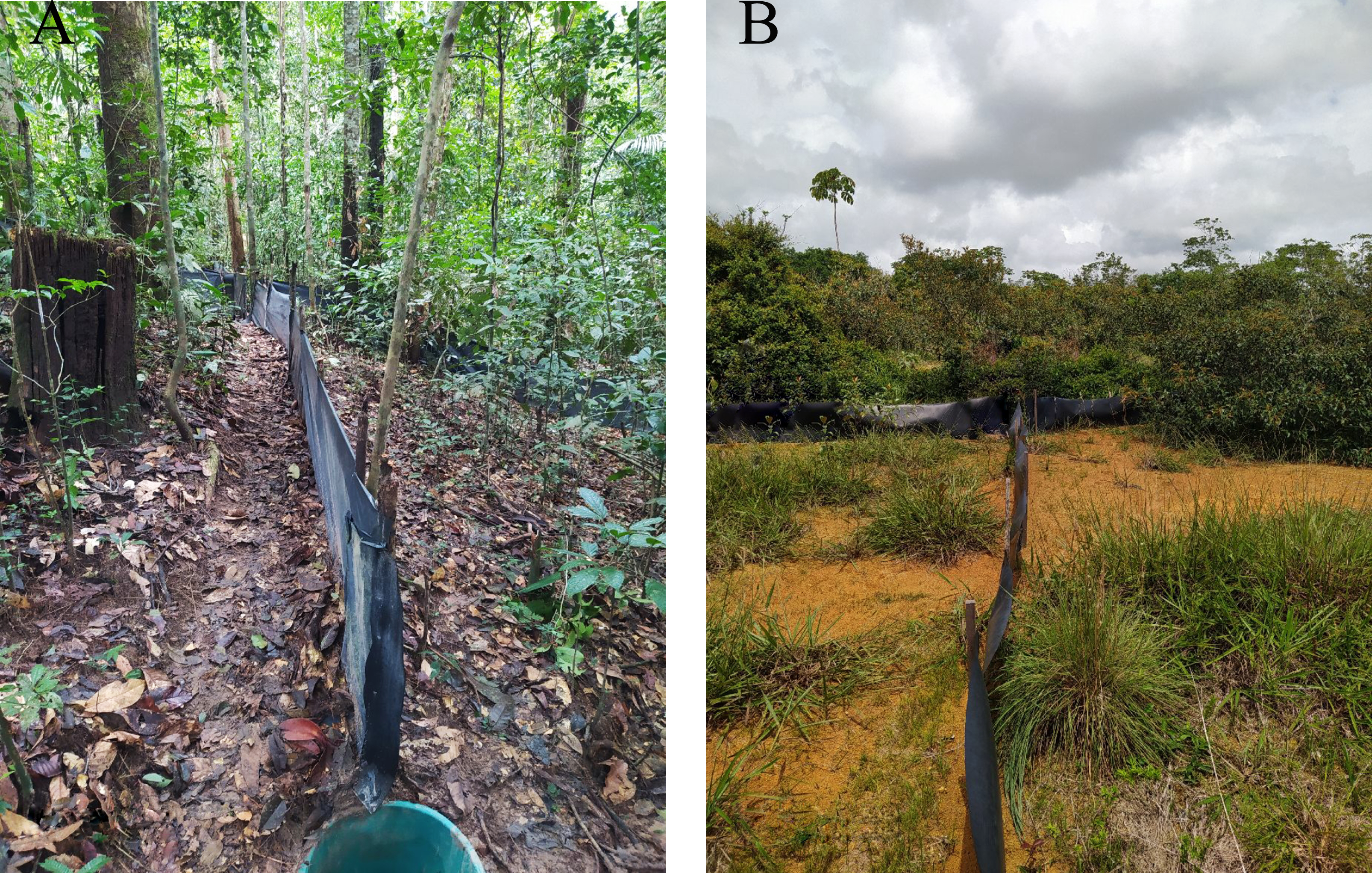

Supplement: S1 Fig — (A) Pitfall Traps installed in Forest Recovery Areas (FRA) and (B) in Altered Primary Forest (APF) areas. (TIFF) [file pone.0315904.s002.tiff]

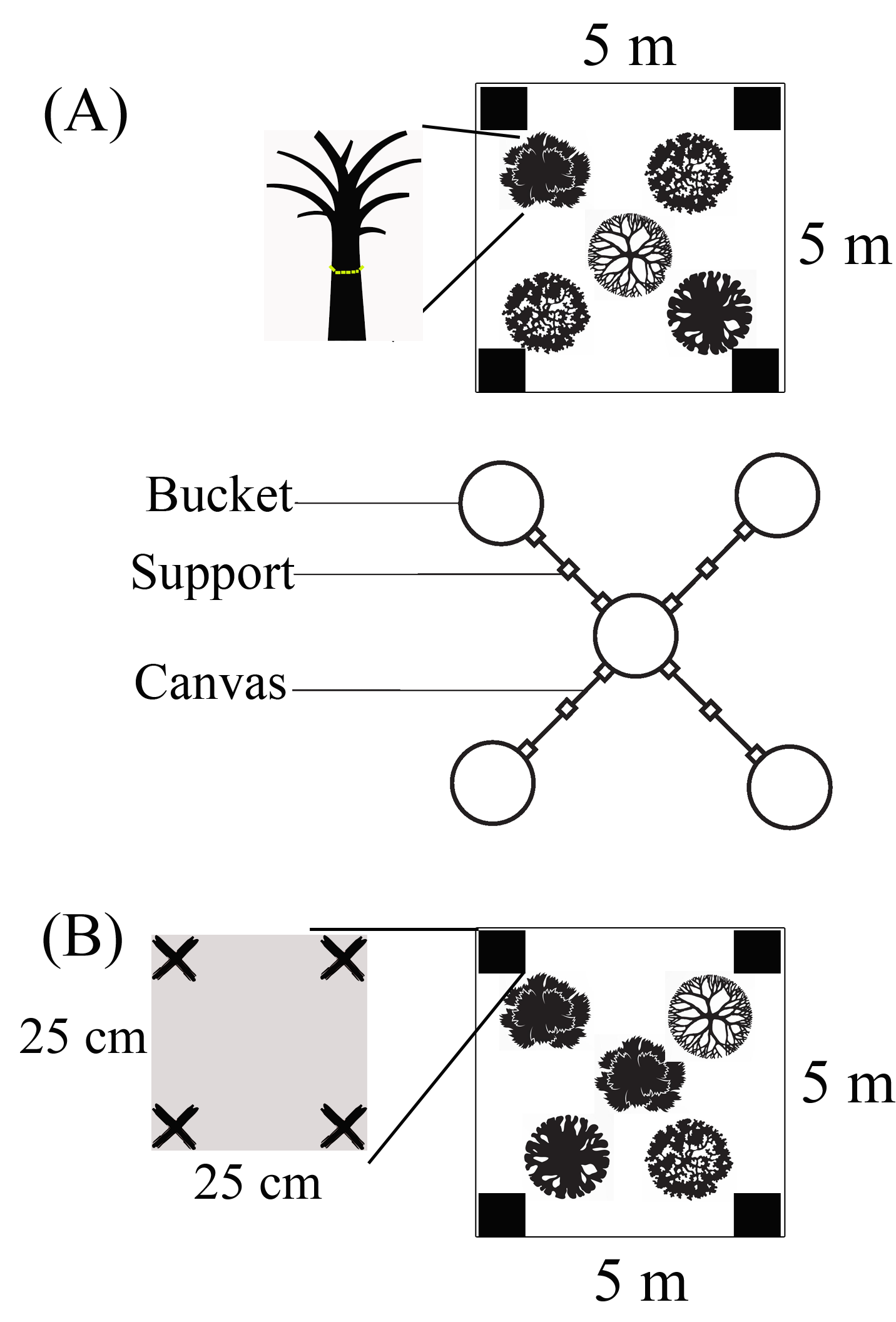

Supplement: S2 Fig — (A) Basal area sampling, in 5 x 5 m plots; (B) Litter height sampling in 25 x 25 cm subplots. (TIFF) [file pone.0315904.s003.tiff]
