# Supplementary material for: Multiparameter analysis of small non-flying mammals’ response to forest restoration post-bauxite mining in eastern Amazonia
Source: PLoS One. 2025 Jan 24;20(1):e0315904. doi: 10.1371/journal.pone.0315904 (PMC11759357; doi:10.1371/journal.pone.0315904)
Supplement: S1 Table — (DOCX) [file pone.0315904.s004.docx]

**S1 Table.** Description of functional traits used for functional analyses, including their measurement units, value categories, and functional meanings.

| **Functional traits** | **Unit of measurement** | **Category** | **Functional meaning** |
| --- | --- | --- | --- |
| Body mass | Grams | Quantitative - Continuous | Specific metabolic demand, risk of predation. |
| Habit | Terrestrial, arboreal, scansorial and semi-aquatic | Categorical_Binary | Use of food and space resources according to biotic and abiotic components. |
| Activity | Day, Evening, Night | Categorical_Binary | Predation and anti-predation strategies, in addition to temporal niche partitioning with other species. |
| Trophic guild | Insectivore (diet greater than or equal to 50% insects), Frugivore (diet greater than or equal to 50% fruits and seeds), Granivore (diet greater than or equal to 50% plants), and Omnivore (diet varies between insects, fruits, nectar, seeds, plants, carrion, small vertebrates and fungi) | Categorical_Binary | Quality and quantity of resources consumed and released by species, thus providing information on ecosystem energy flows. |
