# Supplementary material for: Multiparameter analysis of small non-flying mammals’ response to forest restoration post-bauxite mining in eastern Amazonia
Source: PLoS One. 2025 Jan 24;20(1):e0315904. doi: 10.1371/journal.pone.0315904 (PMC11759357; doi:10.1371/journal.pone.0315904)
Supplement: S2 Table — Habit (locomotion): (Sc) Scansorial, (Ar) Arboreal, (Te) Terrestrial, (Saq) Semi-aquatic; Period of activity: (Noc) Nocturnal, (Cre) Crepuscular, (Day) Daytime, Aci (Acyclic); Trophic guild/diet: (Inv) Invertebrates; (Vend) Mammals and birds; (Vect) Reptiles and amphibians; (Vunk) Vertebrates in general or unidentified (Scav) Organic garbage and carrion, (Fru) Fruit, (Nec) Nectar and pollen, (See) Seeds, spores and grains, (Pla) Grass, seedlings, tubers, bulbs, lichens, moss, roots, twigs, bark and leaves, (Fun) Mycorrhizal fungi; Morphology: (Bod_m) Body mass (in grams). (DOCX) [file pone.0315904.s005.docx]

**S2 Table** - Morphological and functional attributes of the species of rodents and marsupials sampled. Legend, Habit (locomotion): (Sc) Scansorial, (Ar) Arboreal, (Te) Terrestrial, (Saq) Semi-aquatic; Period of activity: (Noc) Nocturnal, (Cre) Crepuscular, (Day) Daytime, Aci (Acyclic); Trophic guild/diet: (Inv) Invertebrates; (Vend) Mammals and birds; (Vect) Reptiles and amphibians; (Vunk) Vertebrates in general or unidentified (Scav) Organic garbage and carrion, (Fru) Fruit, (Nec) Nectar and pollen, (See) Seeds, spores and grains, (Pla) Grass, seedlings, tubers, bulbs, lichens, moss, roots, twigs, bark and leaves, (Fun) Mycorrhizal fungi; Morphology: (Bod_m) Body mass (in grams).

| **Species** | **Habit** | **Activity** | **Trophic guild/diet** | **Body Mass** |
| --- | --- | --- | --- | --- |
| **Didelphimorphia** | | | | |
| *Didelphis marsupialis* | Te/Sc/Ar | Noc | Omnivorous (Inv, Vunk, Scav, Fru, Nec, Pla) | 1351 |
| *Marmosa demerarae* | Ar | Noc | Insectivorous (Inv, Fru, Vend, Nec) | 88.75 |
| *Marmosops marina* | Te/Sc | Noc | Insectivorous and Frugivore (Inv, Fru) | 20.42 |
| *Marmosops pinheroi* | Te/Sc | Noc | Insectivorous and Frugivore (Inv, Fru) | 27 |
| *Marmosops woodalli* | Te/Sc | Noc | Insectivorous and Frugivore (Inv, Fru) | 18 |
| *Monodelphis americana* | Te | Day | Omnivorous (Inv, Vend, Scav, Fru, See) | 54.4 |
| *Philander opossum* | Te/Sc | Noc | Omnivorous (Inv, Fru, Nec, Vend, Vect, Vunk) | 272.25 |
| **Rodentia** | | | | |
| *Calomys tener* | Te | Noc/Twi | Granivore (Pla, Fru) | 20.3 |
| *Echimys chrysurus* | Ar | Noc | Frugivore (Fru, Pla) | 624.99 |
| *Hylaeamys megacephalus* | Te, Sc | Aci | Omnivorous (Inv, Fru, Pla, See) | 55.5 |
| *Hylaeamys yunganus* | Te | Aci | Omnivorous (Inv, Fru, See, Pla) | 40.87 |
| *Makalata didelphoides* | Ar | Noc | Frugivore (Fru, See, Pla) | 340 |
| *Mesomys stimulax* | Ar | Noc | Frugivore, Granivore (Fru, See, Pla) | 141.5 |
| *Necromys lasiurus* | Te | Day | Insectivorous (Inv, See, Pla) | 56.43 |
| *Oecomys* cf. *roberti* | Ar | Noc | Frugivore (Fru, See) | 233.2 |
| *Oecomys* gr. *paricola* | Ar | Noc | Frugivore (Fru, See) | 28.87 |
| *Oecomys* gr. *catherinae* | Ar | Noc | Frugivore (Fru, See) | 73.4 |
| *Oligoryzomys gri apinaye* | Te | Noc | Frugivore (Inv, Fru, See) | 24.16 |
| *Proechimys roberti* | Te | Noc | Granivore (Inv, See, Fru, Pla, Fun) | 284.99 |
| *Pseudoryzomys simplex* | Te/Saq | Noc | Omnivorous (Inv, Fru, See, Pla) | 29.2 |
| *Rhipidomys emiliae* | Ar | Noc | Frugivore (Inv, Fru, See, Pla) | 35.66 |
| *Rhipidomys nitela* | Ar | Noc | Frugivore (Inv, Fru, See, Pla) | 89.49 |
