# Supplementary material for: Multiparameter analysis of small non-flying mammals’ response to forest restoration post-bauxite mining in eastern Amazonia
Source: PLoS One. 2025 Jan 24;20(1):e0315904. doi: 10.1371/journal.pone.0315904 (PMC11759357; doi:10.1371/journal.pone.0315904)
Supplement: S3 Table — Abundance (N) Number of specimens sampled in RFA (Recovery Forest Area) and APF (Altered Primary Forest). (DOCX) [file pone.0315904.s006.docx]

**S3 Table -** The taxonomic structure (composition, richness and abundance) recorded in the small mammal community in the studied areas. Abundance (N) Number of specimens sampled in RFA (Recovery Forest Area) and APF (Altered Primary Forest).

| **Order/species** | **Abundance** | |
| --- | --- | --- |
|  | RFA | APF |
| **Didelphimorphia**  **Didelphidae (Opossuns)** | | |
| *Didelphis marsupialis* | 0 | 1 |
| *Marmosa demerarae* | 0 | 2 |
| *Marmosops marina* | 1 | 14 |
| *Marmosops pinheroi* | 0 | 1 |
| *Marmosops woodalli* | 0 | 2 |
| *Monodelphis americana* | 3 | 5 |
| *Philander opossum* | 1 | 1 |
| **Rodentia**  **Cricetidae (Small rats)** |  |  |
| *Calomys tener* | 10 | 0 |
| *Hylaeamys megacephalus* | 0 | 2 |
| *Hylaeamys yunganus* | 1 | 4 |
| *Necromys lasiurus* | 16 | 0 |
| *Oecomys* cf *roberti* | 1 | 1 |
| *Oecomys* gr. *paricola* | 1 | 6 |
| *Oecomys* gr. *catherinae* | 0 | 1 |
| *Oecomys* sp | 0 | 1 |
| *Oligoryzomys gri apinaye* | 5 | 3 |
| *Pseudoryzomys simplex* | 4 | 5 |
| *Rhipidomys nitela* | 0 | 1 |
| *Rhipidomys emiliae* | 0 | 3 |
| **Echimyidae (Spiny rats)** |  |  |
| *Echimys chrysurus* | 0 | 2 |
| *Makalata didelphoides* | 0 | 1 |
| *Mesomys stimulax* | 0 | 1 |
| *Proechimys roberti* | 0 | 5 |
| Abundance | **43** | **62** |
| Richness | **10** | **22** |
