# Supplementary material for: Multiparameter analysis of small non-flying mammals’ response to forest restoration post-bauxite mining in eastern Amazonia
Source: PLoS One. 2025 Jan 24;20(1):e0315904. doi: 10.1371/journal.pone.0315904 (PMC11759357; doi:10.1371/journal.pone.0315904)
Supplement: S4 Table — (DOCX) [file pone.0315904.s007.docx]

**S4 Table.** Result of the Partial Redundancy Analysis, evaluating the influence of the environment and space in the community.

| **Fraction** | **Partition** | **R2** | **F** | **p** |
| --- | --- | --- | --- | --- |
| [a] | Environmental variables | 0.19 | 1.9223 | 0.017 |
| [b] | Sharing of environmental variables and spatial distribution | 0.11 |  |  |
| [c] | Spatial distribution | 0.09 | 1.5901 | 0.072 |
| [d] | Waste | 0.61 |  |  |
