# Supplementary material for: Multiparameter analysis of small non-flying mammals’ response to forest restoration post-bauxite mining in eastern Amazonia
Source: PLoS One. 2025 Jan 24;20(1):e0315904. doi: 10.1371/journal.pone.0315904 (PMC11759357; doi:10.1371/journal.pone.0315904)
Supplement: S5 Table — (DOCX) [file pone.0315904.s008.docx]

**S5 Table -** Percentage of explanation of the variables for each axis of the Canonical Correlation Analysis.

|  | **CCA1** | **CCA2** |
| --- | --- | --- |
| Basal area | -0.60093 | -0.74440 |
| Litter | -0.84738 | -0.29651 |
| % Forest cover | -0.93722 | -0.02135 |
| Eigenvalue | 0.6535 | 0.3826 |
| % Explanation | 0.4919 | 0.2880 |
| % Accumulated | 0.4919 | 0.7799 |
| Pearson correlation | 0.9484175 | 0.9834213 |
